# Energy Absorption of Curvilinear Hybrid Auxetic Honeycombs

**DOI:** 10.3390/ma19091791

**Published:** 2026-04-28

**Authors:** Siyun Li, Na Qiu, Wei Liu, Jie Yang, Qiang Gao

**Affiliations:** 1 School of Mechanical Engineering, Southeast University, Nanjing 211189, China; 213233534@seu.edu.cn; 2 School of Aerospace, Mechanical and Mechatronic Engineering, The University of Sydney, Sydney, NSW 2006, Australia; nnaqiu@163.com; 3 School of Automotive Engineering, Yancheng Institute of Technology, Yancheng 224051, China; liuwei@ycit.edu.cn; 4 National Automotive Lightweighting (Jiangsu) Automotive Technology Co., Ltd., Yangzhou 225009, China; yangj@sae-china.org

**Keywords:** auxetic honeycomb, energy absorption, mechanical properties, parametric design, multi-objective optimization

## Abstract

Auxetic cellular materials attract increasing attention for crashworthiness and impact protection due to their negative Poisson’s ratio (NPR). However, conventional double-arrowhead auxetic honeycombs (DAHs) with straight ligaments often exhibit limited energy absorption and unstable collapse under large deformation. In this study, a curvilinear hybrid auxetic honeycomb (CHAH) is proposed by replacing straight walls with smoothly curved ligaments and embedding a circular positive Poisson’s ratio subcell to provide symmetric support. The mechanical behavior of the CHAH is investigated through a combined experimental–numerical approach. Finite element simulations are validated by quasi-static compression experiments, and a parametric study is conducted to evaluate the influence of key geometric variables on specific energy absorption (SEA) and peak crushing force (PCF). Based on the validated simulations, a multi-objective optimization framework integrating optimal Latin hypercube sampling, radial basis function surrogate modeling, and NSGA-II is employed to optimize the structural parameters. Compared with the conventional DAH under identical material and volume conditions, the CHAH exhibits significantly improved deformation stability and energy absorption capability, with SEA increasing by up to 67.06% and a more stable plateau response. In addition, SEA and PCF can be effectively tuned by varying the geometric angles (*θ*_1_, *θ*_2_).

## 1. Introduction

Auxetic materials, characterized by lateral expansion under tension and lateral contraction under compression, exhibit exceptional energy absorption capability and have attracted substantial interest in electronics [1], aerospace [2], and biomedicine [3]. First fabricated by Lakes et al. in 1987 [4], auxetic materials offer advantageous mechanical properties, including high shear resistance [5], indentation resistance [6], fracture resistance [7], and enhanced energy absorption [8,9]. Representative auxetic architectures include double-arrowhead structures [10], star-shaped structures [11], chiral structures [12], and rotating-unit structures [13], among others. Accordingly, auxetic structures have been explored for crash-buffering components in automotive engineering [14], lightweight spacecraft structures [15], textiles [16], medical devices [17], and sports equipment [18,19].

As a representative auxetic architecture, the double-arrowhead auxetic honeycomb (DAH) has been widely investigated for energy-absorbing applications owing to its high geometric design flexibility and controllable deformation modes [20,21]. However, double-arrowhead honeycombs, typically composed of straight struts, often exhibit a nearly constant plateau stress with limited energy absorption efficiency. Moreover, they are prone to nonuniform deformation at large strains, which hampers the simultaneous optimization of energy absorption performance and structural stability [22]. Consequently, geometric/topological innovations coupled with advanced manufacturing have become major research directions.

Existing enhancement strategies can be broadly categorized as (i) component addition and (ii) local substitution. Within the component-addition paradigm, embedding self-similar inclusions into re-entrant hexagonal structures improves stiffness and energy absorption capacity [23], while adding a straight rib along the inner major axis of a novel auxetic tube structure markedly increases the specific energy absorption (SEA) [24]. Prior studies also indicate that curvilinear design provides an effective route to improving the mechanical performance of auxetic structures [25]. From the local-substitution perspective, introducing sinusoidal ligaments into a conventional hexagonal honeycomb and adopting a parallel hybridization design yields a three-dimensional sinusoidal–parallel hybrid honeycomb (SPHH), thereby improving the energy absorption and impact-resistance performance compared with the straight-strut baseline [26]. Likewise, incorporating an elliptical annular reinforcement structure (EARE) into a re-entrant honeycomb triggers curvature-induced multistage deformation, increasing the mean plateau stress and SEA by 171.63% and 28.03%, respectively [27]. Nevertheless, prior studies have predominantly focused on re-entrant and rotating star-type topologies, whereas comparatively fewer efforts have addressed the double-arrowhead honeycomb (DAH) family. For DAH-related designs, modifying interfacial contact in a double-arrowhead-assisted honeycomb sandwich from line-to-line to face-to-face contact can improve assembly efficiency [28]. Bioinspired curvilinear redesign of the conventional double-arrowhead geometry, informed by cuttlebone architectures, has enabled strain-tunable honeycomb designs [29]. Furthermore, integrating a diamond honeycomb (DH) into a DAH forms a hybrid D2 honeycomb (D^2^H) with substantially enhanced energy absorption compared with either a DH or DAH alone [30]. Overall, approaches that explicitly couple component addition with local substitution to further improve honeycomb energy absorption remain relatively scarce. In addition, multi-objective optimization is widely employed for structural refinement [31,32]; for example, rearranging DAH units into a cylindrical automotive crash box and optimizing the design can maximize energy absorption without increasing mass [33]. Recent studies have also shown that design-encoded additive manufacturing can achieve programmable structural transformation through anisotropic architectures and controlled printing parameters [8]. Such strategies highlight that manufacturability and process parameters may significantly influence the achievable geometry and structural performance of architected materials.

To address the limited energy absorption efficiency of the DAH [30] and its instability at high strains [19], this study proposes a novel curvilinear hybrid auxetic energy-absorbing architecture (CHAH) [19]. The design prioritizes enhanced energy dissipation [34] and is enabled by a localized topology optimization strategy [35]. First, the straight ligaments in the conventional DAH are replaced by circular arcs [36] to promote progressive, multistage plastic deformation under compression and thereby extend the energy dissipation pathway [37,38]. Second, a circular substructure with a positive Poisson’s ratio is embedded within each curvilinear unit cell [39]; the symmetric support provided by the circular geometry mitigates stress concentration and suppresses nonuniform deformation [40,41]. Through the synergistic interaction between arc-bending deformation and circular back-support [42], the proposed CHAH enhances stable plateau-stage energy absorption, enabling coordinated optimization of energy absorption efficiency and deformation stability. Moreover, while this study primarily focuses on quasi-static energy absorption, the proposed CHAH design is compatible with the future integration of high-fidelity modeling and experimental validation approaches [43], as employed in recent studies on advanced composite and cellular structures, thereby laying the groundwork for more comprehensive investigations of structural reliability and dynamic performance. The remainder of this paper is organized as follows. Section 2 describes the transformation from a DAH to a CHAH via component addition and local substitution and validates the finite element model. Section 3 analyzes CHAH deformation modes and benchmarks its performance against the DAH; it further constructs a parametric library of curvilinear double-arrowhead unit cells [44] and systematically evaluates the effects of geometric parameters—such as aspect ratio, half-cell width, and the angle between the long/short beams and the axis—on mechanical response [45]. Section 4 applies a genetic algorithm to conduct multi-objective optimization and identify the optimal geometric parameter set [46,47] to identify the optimal set of geometric parameters.

## 2. Experimental Setup and Finite Element Model

**Figure 1:**
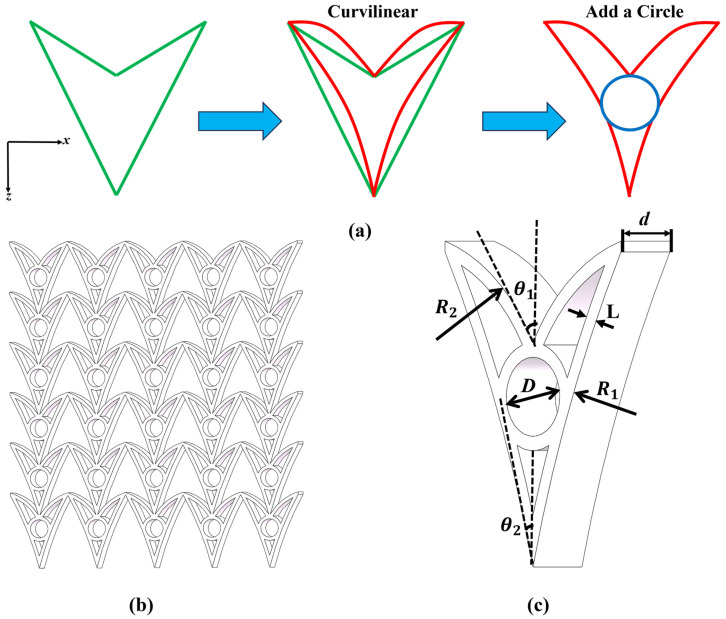
Configuration and geometric structure of the design: (**a**) design strategy of the model; (**b**) solid model of the curvilinear hybrid auxetic honeycombs; (**c**) geometric schematic of the honeycomb model.

Curvilinear hybrid auxetic honeycombs (CHAHs) are obtained by curving the walls of a DAH and introducing a circular motif with a positive Poisson’s ratio. Figure 1 illustrates the design strategy and periodic architecture of the proposed model. The structure is symmetric about the horizontal and vertical axes and is centrosymmetric.

### 2.1. Structural Design

Figure 1a illustrates the geometric design strategy for the initial CHAH unit cell proposed in this study. The unit cell geometry is defined by four independent variables, as shown in Figure 1c. Specifically, *θ*_1_ denotes the angle between the tangent of the upper curved ligament and the axial direction. *θ*_2_ denotes the corresponding angle for the lower curved ligament. The geometric shape of the curved ligaments is further characterized by two curvature radii, *R*_1_ and *R*_2_, which describe the radii of the upper and lower curved beams, respectively, and determine the curvature profile of the ligaments, thereby influencing the overall geometric configuration of the unit cell. In addition, a circular subcell with diameter *D* is embedded at the center of the unit to provide symmetric support during deformation. *d* is the out-of-plane thickness, and *L* is the cell wall thickness. In each FE model, the structure comprises *N*_x_ × *N*_z_ unit cells, where *N*_x_ and *N*_z_ denote the numbers of cells along the x and z directions, respectively. Figure 1b shows the initial CHAH model with *N*_x_ × *N*_z_ = 6 × 5. The initial unit cell parameters are *θ*_1_ = 30°, *θ*_2_ = 15°, *R*_1_ = 180 mm, *R*_2_ = 30 mm, *r* = 11 mm, *d =* 10 mm, and *L* = 2 mm. The geometric parameters and their corresponding ranges used in this study are summarized in Table 1.

### 2.2. Finite Element Model

**Figure 2:**
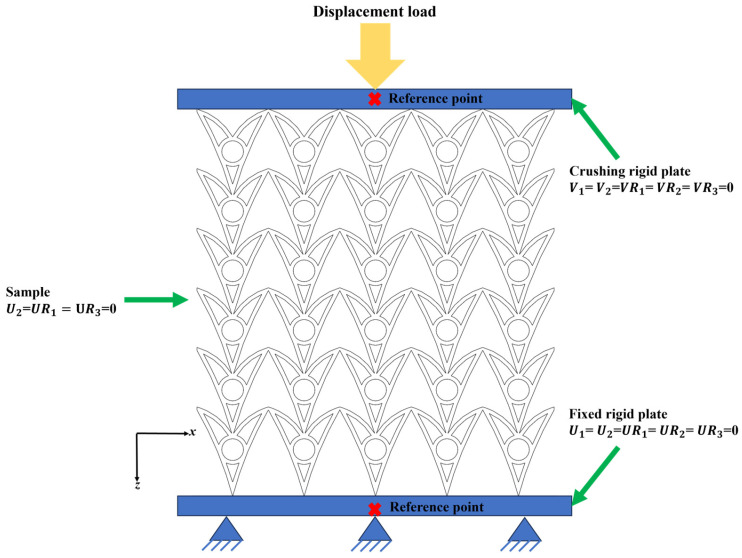
FE model for quasi-static compression with boundary conditions.

**Figure 3:**
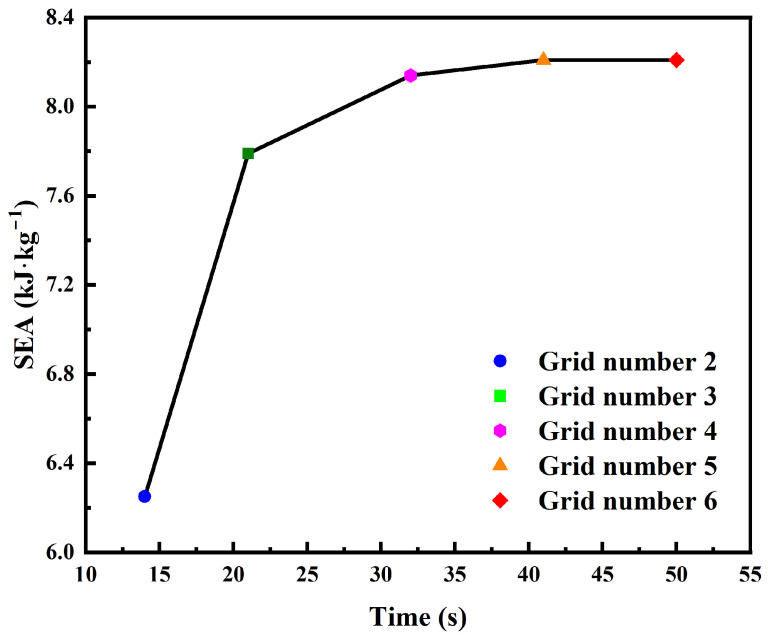
Mesh convergence analysis of the FE model, with the initial CHAH as a representative case. A mesh number of 4 was selected by balancing computational cost and accuracy.

The honeycomb structure is constructed by periodically repeating the unit cell along the x and z directions, with *N*_x_ = 6 and *N*_z_ = 1–6. It should be noted that the finite model contains boundary cells that may introduce slight boundary effects. However, since the structure consists of multiple repeating unit cells, the overall deformation response is mainly governed by the interior cells. Therefore, the influence of boundary effects on the calculated specific energy absorption is considered minimal. Finite element analyses were performed in Abaqus (Version 2021, Dassault Systèmes, Vélizy-Villacoublay, France) to validate the experimental results and conduct numerical simulations. Figure 2 shows the FE model (*N*_x_ × *N*_z_ = 6 × 5), consisting of the upper and lower rigid plates and the CHAH sample. *V*_1_, *V*_2_, and *V*_3_ denote the translational velocity components of the plate/sample along the x-, y-, and z-axes, respectively. *U*_1_, *U*_2_, and *U*_3_ denote the translational displacement components of the plate/sample along the x-, y-, and z-axes, respectively. *VR*_1_, *VR*_2_, and *VR*_3_ denote the angular velocity components of the plate/sample about the x, y, and z axes, respectively. *UR*_1_, *UR*_2_, and *UR*_3_ denote the angular displacement components (rotational displacements) of the plate/sample about the x, y, and z axes, respectively. In this study, periodic boundary conditions (PBCs) were not applied. Instead, the boundary conditions were defined to replicate the finite-sized experimental specimen compressed between two rigid plates, ensuring consistency between the numerical simulations and the quasi-static compression experiments. The boundary conditions were applied as follows. Large deformations and contact nonlinearity were included [48]. Two discrete rigid plates were placed at the top and bottom of the structure to represent the crossheads used in the experiments. Loads and boundary conditions were applied at the reference points of the rigid plates. The upper plate was driven downward at a quasi-static velocity, whereas the lower plate was fully fixed. The honeycomb sample was clamped between the two steel plates. In the general contact definition, hard contact was used in the normal direction, and a penalty formulation was used in the tangential direction. A friction coefficient of μ = 0.3 was used for the contact interaction, which is consistent with commonly reported values for steel–steel contact in previous studies [49]. To improve computational efficiency, the quasi-static compression simulations were conducted using the explicit dynamic solver with mass scaling. This approach is widely adopted in nonlinear FE analyses involving complex contact and large deformation, provided that inertial effects remain negligible and the kinetic energy is sufficiently small compared with the internal energy [50]. The mesh convergence study (Figure 3) shows that the SEA obtained with a 0.5 mm mesh (grid number 4) agrees closely with that obtained with a 0.4 mm mesh (grid number 5). Considering the balance between accuracy and computational cost, a mesh size of 0.5 mm was selected; therefore, the initial model contained four elements through the thickness. The model employed reduced-integration eight-node brick elements (C3D8R). All ligaments were meshed with at least four elements through the thickness to mitigate shear locking [51]. The material behavior of the honeycomb was modeled using an elastic–plastic constitutive law. The base material constituting the honeycomb was assumed to be 316 L stainless steel fabricated by selective laser melting, with density *ρ* = 7.85 g/cm^3^, Poisson’s ratio *ν* = 0.29, Young’s modulus *E*_s_ = 168 GPa, and yield strength *σ*_ys_ = 556 MPa, according to the material datasheet provided by the manufacturer. The corresponding material properties are summarized in Table 2. These material parameters were consistent with those used in the experimental tests. In this study, the material behavior was modeled under quasi-static conditions, and strain rate sensitivity was not considered because the experiments were conducted at a low loading rate (2 mm/min).

### 2.3. Validation of the FE Model

**Figure 4:**
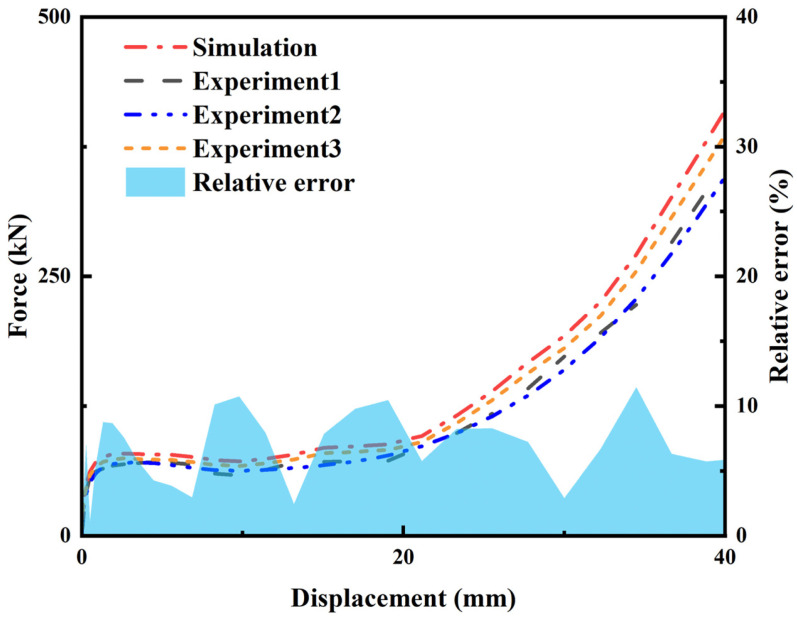
Experimental and FE simulated force–displacement curves under quasi-static loading, along with the relative error between the FE prediction and the mean experimental response.

**Figure 5:**
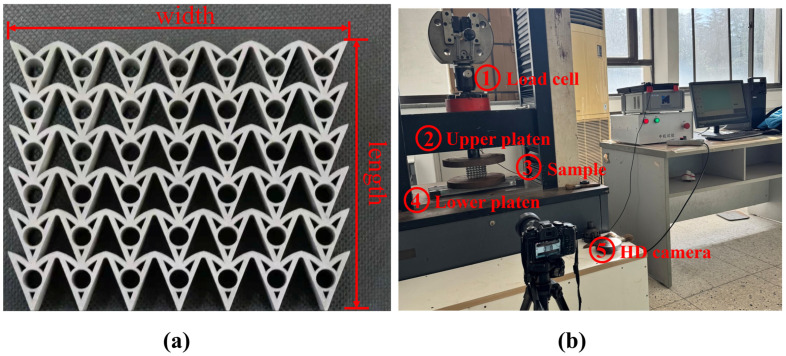
(**a**) CHAH sample fabricated by selective laser melting (SLM). (**b**) Experimental setup for quasi-static uniaxial compression testing.

Figure 4 shows the experimental and FE-simulated force–displacement curves under quasi-static loading, along with the relative error between the FE prediction and the mean experimental response. To validate the finite element (FE) model, corresponding experiments were conducted. As shown in Figure 5a, the sample measured 80 mm in length, 100 mm in width, and 10 mm in thickness. The honeycomb consisted of *N*_x_ × *N*_z_ = 7 × 6 unit cells, with geometric parameters *θ*_1_ = 40°, *θ*_2_ = 20°, *R*_1_ = 272 mm, *R*_2_ = 13 mm, *r* = 4.5 mm, and *L* = 1.5 mm. The experiments were conducted using the same material properties as those adopted in the finite element simulations. These properties were provided by the manufacturer Wenext (Shanghai) Intelligent Technology Co., Ltd., Shanghai, China. The material behavior was described using an isotropic elastic–plastic constitutive model with von Mises yield criterion. Strain hardening was incorporated using the true stress–strain curve obtained from the material datasheet. The sample was manufactured by selective laser melting (SLM) [52].

Subsequently, a quasi-static compression test was performed on the fabricated honeycomb sample using a universal testing machine (Figure 5b). The sample was placed on a fixed steel plate. During testing, the indenter plate moved downward along the axial direction at a constant rate of 2 mm/min. To protect the apparatus, the machine was programmed to stop automatically when the load reached 2000 kN, indicating that the honeycomb was approaching densification. Sample deformation was recorded using a camera.

**Figure 6:**
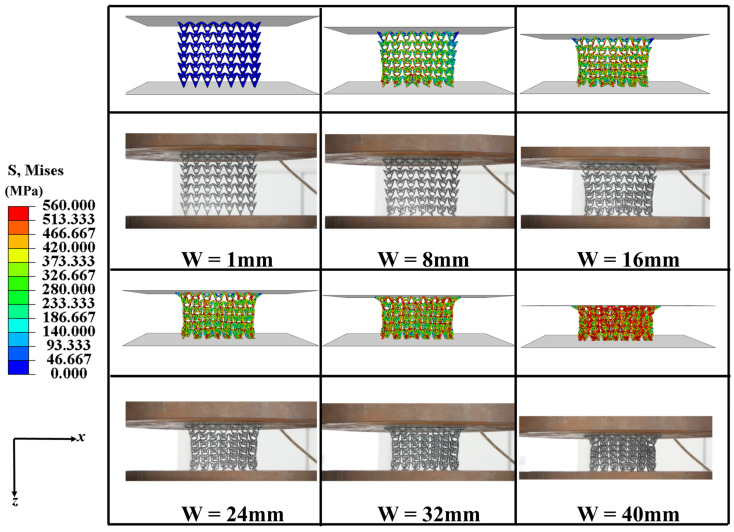
FE model validation. Comparison of deformation modes: experiments vs. FE simulations.

Figure 4 and Figure 6 compare the deformation patterns and force–displacement curves from the experiments and the corresponding FE simulations. The force–displacement curves and deformation modes agree closely between the experiments and the FE simulations.

To quantitatively evaluate the accuracy of the numerical model, the relative error between the simulated and experimental forces is calculated using Equation (1):

(1) $$\mathrm{Error}=\frac{\left|F_{\mathrm{FE}}-F_{\exp}\right|}{F_{\exp}}\times 100\%$$

where *F*_FE_ and *F*_exp_ represent the forces obtained from the FE simulation and the experiments, respectively.

The experimental force *F*_exp_ is calculated using Equation (2):

(2) $$F_{\exp}=\frac{F_{\exp1}+F_{\exp2}+F_{\exp3}}{2}$$

The calculated results show that the mean relative error over the entire compression process is 5.28%. The relatively small discrepancies indicate that the FE model can effectively reproduce the compressive response and deformation characteristics of the structure. Therefore, the proposed FE model is considered sufficiently accurate for subsequent parametric studies and performance analyses.

## 3. Results and Discussion

### 3.1. Evaluation Indicator

The peak crushing force (PCF) is defined as the first pronounced peak in the force–displacement curve, occurring when the applied load first exceeds the yield strength and localized plastic deformation initiates.

To systematically evaluate the energy absorption performance of the CHAH and DAH, the following metrics were used: energy absorption (EA), specific energy absorption (SEA), peak crushing force (PCF), and average bearing force (ABF). The total energy absorption (EA) under quasi-static compression is given by Equation (3):

(3) $$\mathrm{EA}=\int_{0}^{d}Fd\delta$$

where *F* is the compressive load and *d* is the densification displacement of the honeycomb. The energy absorption process was assumed to terminate once densification occurred. The determination of the densification displacement has been widely discussed in the literature [53]. In this study, the densification displacement was defined as the displacement at the strain corresponding to the last local maximum in the energy absorption efficiency–strain curve [54].

Accordingly, the energy absorption efficiency (*η*_δ_) is defined as Equation (4):

(4) ηδ=∫0δF(δ)dδF(δ),dF(δ)dδ δ=d

The energy absorbed per unit mass of the structure, i.e., the specific energy absorption (SEA), is calculated using Equation (5):

(5) $$\mathrm{SEA}=\frac{\mathrm{EA}}{m}$$

where *m* is the mass of the honeycomb. The average bearing force (ABF) reflects the effective stiffness of the structure; a higher ABF indicates a stiffer response. ABF is calculated using Equation (6):

(6) $$\mathrm{ABF}=\frac{\mathrm{EA}}{d}$$

The crushing force efficiency (CFE) characterizes the load uniformity of the honeycomb, as reflected by the force–displacement curve. It is a key metric related to peak stress and specific energy absorption [55]. CFE is calculated using Equation (7):

(7) $$\mathrm{CFE}=\frac{\mathrm{ABF}}{\mathrm{PCF}}$$

### 3.2. Deformation Mode Analysis of the CHAH

**Figure 7:**
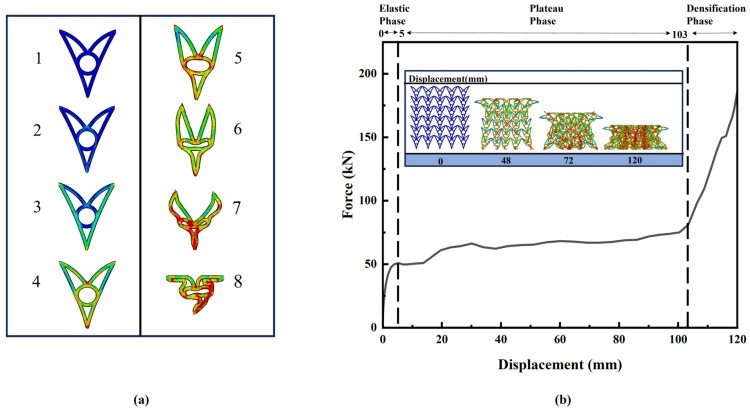
(**a**) Representative cells at selected stages. (**b**) Compressive force–displacement curve of the CHAH and deformation snapshots of the CHAH under compression.

A representative CHAH configuration with parameters *θ*_1_ = 15°, *θ*_2_ = 30°, *d* = 10 mm, and *N*_x_ × *N*_z_ = 6 × 5 was selected to investigate the deformation mechanism under quasi-static compression. The corresponding deformation patterns and force–displacement curve are illustrated in Figure 7. Similar to conventional cellular energy-absorbing structures, the compressive response can be divided into three characteristic stages, namely, the elastic stage, plateau stage, and densification stage [56].

In the elastic stage, the apex corners of the unit cell first contact the internal circular feature, generating localized squeezing interaction accompanied by slight elastic deformation. Most unit cells remain in a stable configuration corresponding to Typical State 2. During this stage, the deformation of the cell walls is dominated by elastic bending, and the macroscopic stress increases approximately linearly with displacement. As the displacement increases to approximately *w* = 5 mm, localized plastic deformation initiates near the nodal regions where stress concentration occurs. Specifically, the unit cell (Typical State 2) shows that the upper and lower curved beams begin to bend elastically while the apex first contacts the internal circular subcell. This circular subcell provides symmetric support, delaying the onset of global instability and promoting uniform energy absorption. Plastic hinges form at the nodal regions, while the curved beams bend progressively under the subcell’s constraint, ensuring a stable plateau-stage response. This event produces the first peak in the force–displacement curve and marks the transition from the elastic regime to the plateau regime. The transition from the elastic stage to the plateau stage is characterized by the onset of local plastic deformation concentrated at the nodal regions. This localized yielding relaxes the stress in the initially loaded areas, causing a small drop after the first peak, before the structure reaches a relatively stable stress level in the plateau regime. The plateau stress is thus governed by progressive plastic bending of the curved beams and load redistribution among the unit cells, rather than by purely elastic response. The early yielding mainly results from the geometric constraint imposed by the circular feature, which amplifies stress concentration at the cell wall nodes through localized contact interaction.

In the plateau stage, plastic zones progressively develop around the nodal regions, and the curved cell walls undergo pronounced plastic bending while the global structure maintains a relatively stable deformation pattern. As the upper curved beam continues to move downward, it compresses the internal circular feature. The circular feature initially deforms elastically and provides additional support to the upper beam. Owing to the geometric symmetry of the circular structure, the reaction force generated during its deformation resists further downward deflection of the curved beam, thereby increasing the stress level during the early plateau stage.

With further compression, the interaction between the curved beams and the circular feature becomes increasingly pronounced. When the displacement reaches approximately *w* = 36 mm, an X-shaped deformation band forms along the diagonal direction of the unit cell array in the FE model of the CHAH structure. This phenomenon indicates a redistribution of the load path within the cellular structure, which is commonly observed in periodic lattice systems subjected to compression [57]. At this stage, the deformed circular feature comes into contact with the lower curved beam, restricting further deformation of the circular structure and transferring the load to the lower beam. This interaction induces plastic bending of the lower curved beam, corresponding to the circular–lower-beam contact deformation mode (Typical State 6). As compression continues, the circular features in most unit cells gradually exceed their elastic limit and begin to deform plastically. Meanwhile, the contact region between the circular feature and the lower curved beam expands, forming a distributed circular–beam support system throughout the structure. This support mechanism promotes the formation of multiple plastic hinges within the cell walls. Because the formation of plastic hinges dissipates substantial plastic work, the energy absorption increases approximately linearly with displacement during the plateau stage [58]. Meanwhile, the stress level rises slightly as the structural support progressively strengthens. Consequently, the deformation state evolves into Typical State 7, indicating that the structure enters a highly efficient energy absorption regime.

In the densification stage, when the displacement exceeds approximately *w* = 77 mm, the unit cells progressively lose their load-carrying capacity and begin to collapse. The cell walls contact and stack against each other, resulting in a rapid reduction in the internal void space. During this densification process, the compressive stiffness of the structure increases significantly, and the stress rises rapidly with further deformation until the structure becomes fully compacted.

From a structural design perspective, the influence of the curvature radius on stress distribution can be qualitatively understood through a simplified beam theory approach. Each curved beam can be approximately considered as a cantilever subjected to bending. According to classical beam theory, the bending stress can be expressed as

(8) $$\sigma =\frac{My}{I}$$

where *M* is the bending moment, *y* is the distance from the neutral axis, and *I* is the second moment of area of the beam cross-section. For a curved beam with curvature radius *R*, the bending moment induced by deformation can be approximately related to the curvature change as

(9) $$M\approx EI\kappa \approx \frac{EI}{R}$$

where *E* is the Young’s modulus and *κ* is the curvature. Combining the above relations indicates that the bending stress is approximately proportional to the curvature, i.e.,

(10) $$\sigma \propto \frac{Ey}{R}.$$

Therefore, the maximum stress decreases as the curvature radius increases. Consequently, introducing a smooth transition with a larger or gradually varying curvature radius reduces local stress concentrations in the nodal regions by distributing the load more evenly along the beam. This design principle explains the FE observations, in which curved beams with smooth transitions form plastic hinges more uniformly, delay local yielding, and contribute to a stable plateau-stage response, thereby enhancing the overall energy absorption efficiency of the CHAH structure.

### 3.3. Comparison with the Conventional Double-Arrowhead Honeycomb (DAH)

**Figure 8:**
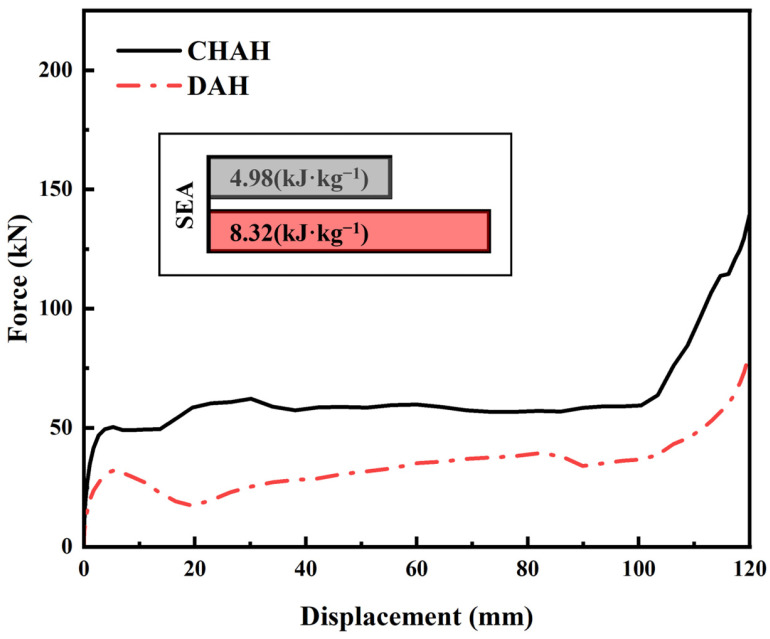
Force–displacement curves of the CHAH and DAH structures.

A DAH sample with *N*_x_ × *N*_z_ = 6 × 5 was constructed with overall dimensions of 180 mm in the x-direction and 195 mm in the z-direction. FE-based compression simulations were then performed for comparison with the reference honeycomb. To assess the energy absorption performance of the CHAH, a systematic comparison was conducted with the conventional double-arrowhead honeycomb (DAH) at the same relative density, in terms of compressive response and energy absorption efficiency. As shown by the force–displacement curves and the specific energy absorption (SEA) in Figure 8, the improved architecture provides higher load-carrying capacity, a more stable plateau response, and higher energy absorption efficiency.

In terms of load-carrying capacity, the force–displacement curve of the improved configuration remains above that of the conventional DAH. In the initial stage (*w* < 20 mm), the peak force approaches 50.38 kN, compared with 32.05 kN for the conventional DAH. The load then remains at a relatively high level until it rises sharply in the densification regime, indicating higher compressive stiffness. In contrast, the conventional DAH shows a lower initial peak and larger force fluctuations, suggesting reduced load-bearing capability.

Regarding energy absorption stability, the improved configuration exhibits a flatter plateau with smaller oscillations. Over 20–100 mm displacement, the load remains above 50,000 N, enabling more uniform energy dissipation. By contrast, the conventional DAH shows distinct load drops and abrupt force changes, indicating a less stable energy absorption process and reduced cushioning performance.

For specific energy absorption (SEA), the improved configuration reaches 8.32 kJ·kg^−1^, which is 1.67 times that of the conventional DAH (4.98 kJ·kg^−1^). This result indicates that, for the same mass, the improved configuration absorbs more energy and therefore achieves higher energy absorption efficiency. Overall, the structurally optimized honeycomb outperforms the conventional DAH in load capacity, plateau stability, and energy absorption efficiency, making it promising for applications requiring high energy absorption (e.g., automotive crashworthiness and protective equipment) [59].

### 3.4. Parametric Analysis

The double-arrowhead honeycomb (DAH) has been used in automotive energy-absorbing devices. For example, Zhou et al. incorporated the DAH as the core of a conventional energy absorber, which improved the device’s energy absorption capacity [60]. Based on the comparative experiments in Section 2.1, the curved double-arrowhead honeycomb (CHAH) proposed in this study shows improved energy absorption performance and is therefore a promising candidate for energy-absorbing devices. In Section 3.4, a finite element analysis (FEA) framework is used to investigate how CHAH multiscale parameters influence its energy absorption behavior. In this section, the effects of the key parameters *N*_x_ × *N*_z_, *d*, *θ*_1_, and *θ*_2_ on the mechanical performance of the CHAH are systematically examined. Other geometric variables are kept constant to isolate their influences. Specifically, the curvature radii are fixed at *R*_1_ = 180 mm and *R*_2_ = 30 mm, and the cell wall thickness is set to *L* = 2 mm. The circular subcell diameter D varies with the geometric configuration and is therefore not considered as an independent design variable in the present analysis.

#### 3.4.1. Effect of N_x_ × N_z_

**Figure 9:**
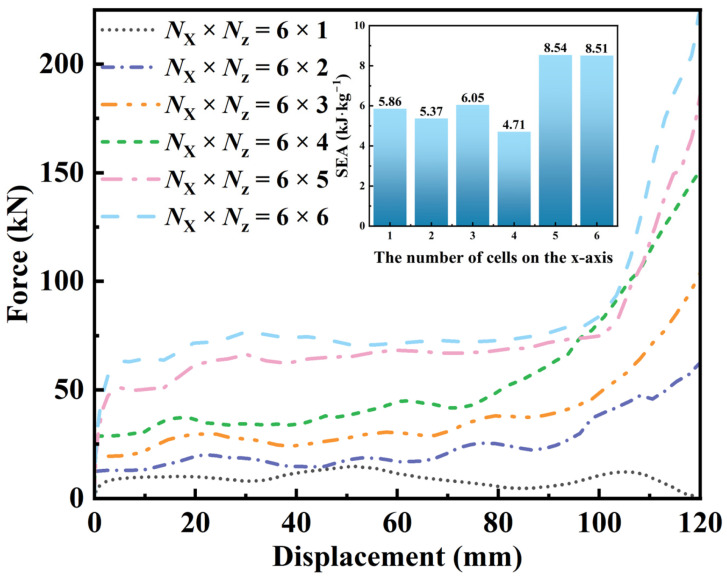
Mechanical properties of honeycomb structures with different *N*_x_ × *N*_z_ configurations.

To examine how the unit cell count affects energy absorption and deformation modes, five configurations with different cell counts were simulated in Abaqus. Figure 9 shows the force–displacement curves and specific energy absorption (SEA) of the proposed honeycomb with different cell counts. The 6 × 5 configuration shows the highest SEA among the cases considered. As the cell count increases, SEA approaches a plateau, suggesting that boundary effects are more pronounced when the cell count is below 6 × 6. The corresponding deformation modes are shown in Figure 9. When the cell count is below 6 × 5, the limited number of central concavities amplifies boundary effects [61]. In contrast, when the cell count exceeds 6 × 6, no substantial differences in deformation mode are observed. Under the additive manufacturing constraints, a trade-off is required between cell count (representativeness) and sample size and cost. Therefore, the 6 × 5 configuration is used throughout this study, and the macroscopic dimensions in the x-*z* plane are set to 180 × 195 mm.

#### 3.4.2. Effect of d

**Figure 10:**
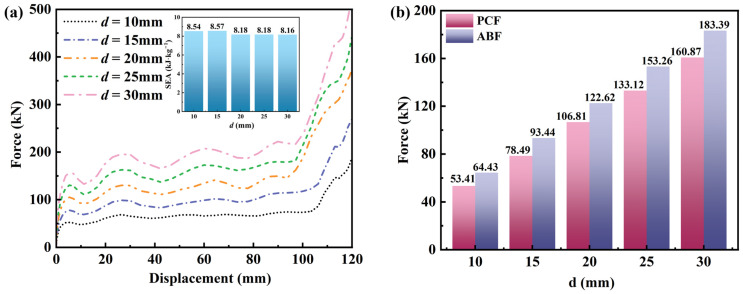
Mechanical performance of honeycomb structures with different wall thicknesses: (**a**) force–displacement curves; (**b**) bar chart of PCF/ABF.

Five honeycomb samples with different thicknesses (*d* = 10.0, 15.0, 20.0, 25.0, and 30.0 mm) were designed. To isolate the effect of thickness, *θ*_1_, *θ*_2_, and *N*_x_ × *N*_z_ were fixed at 30°, 15°, and 6 × 5, respectively, for all samples. The macroscopic dimensions in the x-z plane were set to 180 × 195 mm. Figure 10 illustrates the deformation patterns of the honeycombs with different *d* values under quasi-static compression. All five honeycombs exhibit a pronounced negative Poisson’s ratio (NPR) effect. Across the deformation process, the responses remain similar at the same compression levels. Specifically, all samples undergo the same contraction stage. When the compressive displacement reaches 48 mm, all FE models develop a distinct X-shaped deformation band (Figure 10). The band locations are essentially identical across the models. This consistent deformation mode likely contributes to the similar SEA values.

**Figure 11:**
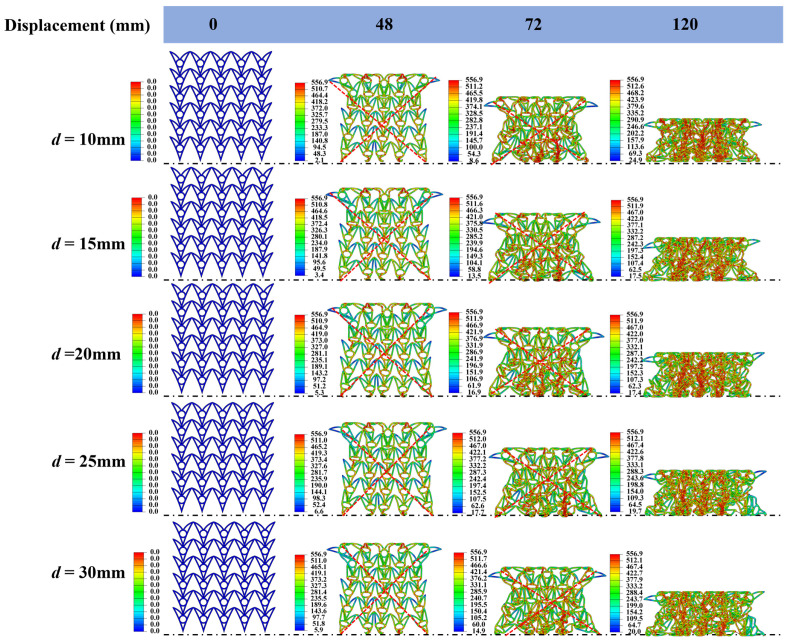
Deformation patterns of honeycomb structures with different d values under quasi-static compression (the stress unit in this figure is MPa).

Figure 11 summarizes the force–displacement curves, SEA, PCF, and ABF. PCF increases approximately linearly with *d*, rising from 53.41 kN at *d* = 10.0 mm to 183.39 kN at *d* = 30.0 mm (243.4% increase). Similarly, ABF increases from 51.55 kN at *d* = 10.0 mm to 109.14 kN at *d* = 30.0 mm (111.7% increase). This trend is expected because increasing *d* increases the second moment of area of the cell walls, thereby improving resistance to plastic deformation [19].

As *d* increases from 10.0 to 30.0 mm, SEA decreases slightly from 8.54 to 8.18 kJ·kg^−1^ at *d* = 20.0 mm and then remains nearly constant. This stability is primarily attributable to the constant relative density. In this study, the ratio of cell wall thickness to cell size is kept identical for all samples, resulting in an unchanged relative density. Because SEA depends mainly on relative density and deformation mode, it remains approximately constant when both are unchanged.

#### 3.4.3. Effect of θ_1_

**Figure 12:**
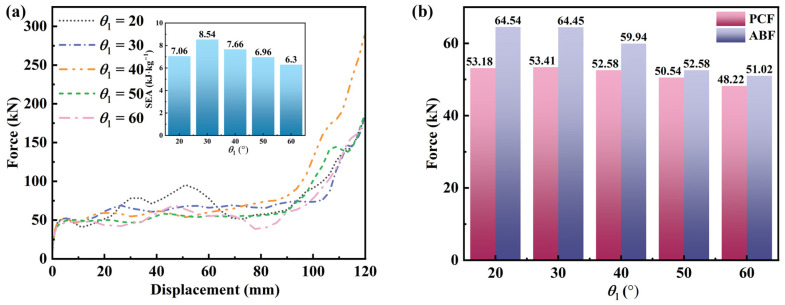
Mechanical performance of honeycomb structures with different *θ*_1_ values: (**a**) force–displacement curves; (**b**) bar chart of PCF/ABF.

**Figure 13:**
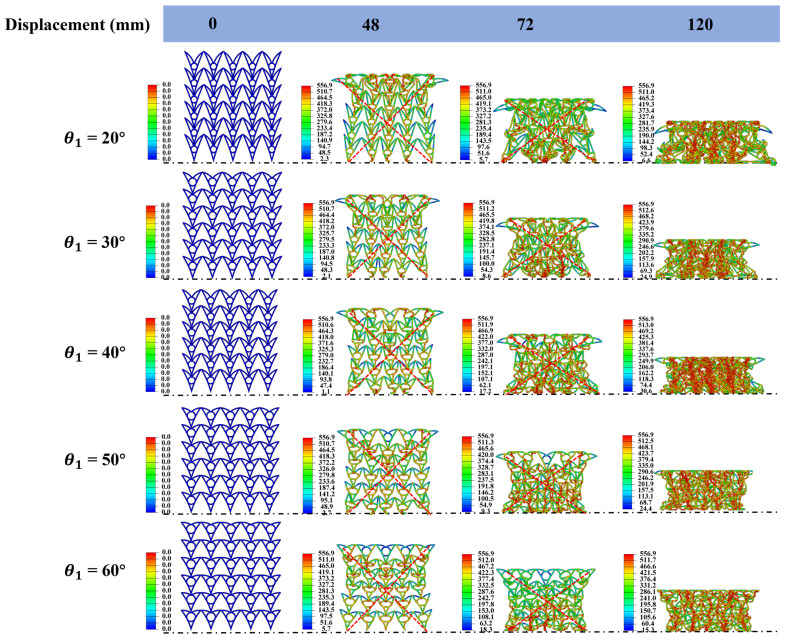
Deformation patterns of honeycomb structures with different *θ*_1_ values under quasi-static compression (the stress unit in this figure is MPa).

Five honeycomb samples with different θ_1_ values (20°, 30°, 40°, 50°, and 60°) were designed. To isolate the effect of θ_2_, d, and N_x_ × N_z_ were fixed at 15°, 10 mm, and 6 × 5, respectively, for all samples. The macroscopic dimensions in the x-z plane were 180 × 195 mm. Figure 12 summarizes the force–displacement curves and related force metrics for different θ_1_ values. Figure 13 shows the deformation patterns for different θ_1_ values under quasi-static compression. All five configurations exhibit a pronounced negative Poisson’s ratio (NPR) effect [62]. At a compressive displacement of 48 mm, all five samples develop a characteristic X-shaped deformation band. At this stage, the curved beams deform cooperatively with the central circular feature, and no obvious local instability is observed. For θ_1_ = 20–30°, the upper curved beams have a moderate inclination, providing well-matched support and guidance to the central circular feature. The compressive load is distributed more uniformly, and the structure deforms globally without pronounced local stress concentrations. The cell beams deform consistently, with minimal torsion or eccentric loading. At θ_1_ = 40°, stability decreases slightly and minor local load nonuniformity emerges; however, global cooperative deformation is maintained. In contrast, for θ_1_ = 50° and 60°, the upper curved beams become overly inclined and provide weaker support. The structure then tends to deform nonuniformly, with delayed deformation in the upper region and concentrated deformation in the lower region. Slight torsion of the cell beams may also occur, indicating progressive deterioration in deformation stability when θ_1_ > 30° [21].

PCF decreases gradually as θ_1_ increases. As θ_1_ increases from 20° to 60°, PCF decreases from 53.18 kN to 48.22 kN (≈9.3% reduction), indicating a mild change. This trend is attributed to the increased inclination of the upper curved beams, which weakens their vertical support for the central circular feature and slightly reduces the initial peak load. However, the radius of the central circular feature increases with θ_1_ to maintain a constant cell size, which partially offsets the loss of support; consequently, the reduction in PCF remains limited. ABF decreases substantially as θ_1_ increases. When θ_1_ increases from 20° to 60°, ABF decreases from 64.54 kN to 51.02 kN (≈21.0% reduction), which is substantially larger than the PCF change. This difference is expected because ABF reflects the load-carrying capacity during the plateau stage. Increasing θ_1_ weakens the support provided by the upper curved beams and alters the load transfer path. More inclined beams produce larger lateral force components, which reduces the effective vertical load-bearing capacity. Although the enlarged central radius provides additional support, it does not fully compensate for the load loss caused by the altered load transfer path, resulting in a pronounced reduction in ABF.

For *θ*_1_ = 20–30°, SEA increases from 7.06 kJ·kg^−1^ to 8.54 kJ·kg^−1^, peaking at 30°. In this range, increasing *θ*_1_ improves the interaction between the upper curved beams and the central circular feature, which enhances deformation stability and increases the energy absorbed during the plateau stage. In addition, the cell wall mass changes only slightly with *θ*_1_ because the central radius increases modestly; therefore, SEA increases as the absorbed energy increases. For *θ*_1_ = 30–60°, SEA decreases monotonically from 8.54 kJ·kg^−1^ at 30° to 6.3 kJ·kg^−1^ at 60°. In this regime, further increases in *θ*_1_ enlarge the central radius and can enhance deformation-based dissipation. However, this benefit does not offset the loss caused by reduced load capacity, particularly the decrease in ABF. Lower ABF reduces the energy absorbed per unit displacement in the plateau stage, and reduced stability promotes earlier local instabilities, which further degrade energy absorption efficiency and lead to a sustained decrease in SEA.

The increase in SEA from 20° to 30°, followed by a decrease from 30° to 60°, indicates an optimal range of *θ*_1_ (20–30°). Within *θ*_1_ = 20–30°, the structure achieves high energy absorption efficiency and the most stable deformation, yielding the highest energy absorbed per unit mass without apparent local instability. For *θ*_1_ < 20°, load capacity may remain relatively high; however, overly strong support can promote premature local failure, limiting further gains in SEA. For *θ*_1_ > 30°, SEA decreases with increasing angle and deformation stability deteriorates, which may not meet engineering requirements for stable and efficient energy absorption.

#### 3.4.4. Effect of θ_2_

**Figure 14:**
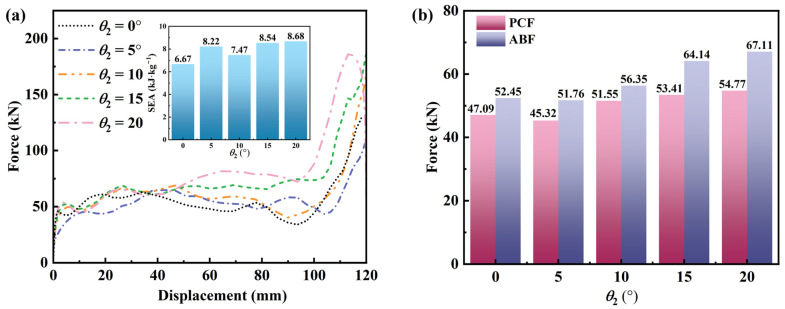
Mechanical performance of honeycomb structures with different *θ*_2_ values: (**a**) force–displacement curves; (**b**) bar chart of PCF/ABF.

**Figure 15:**
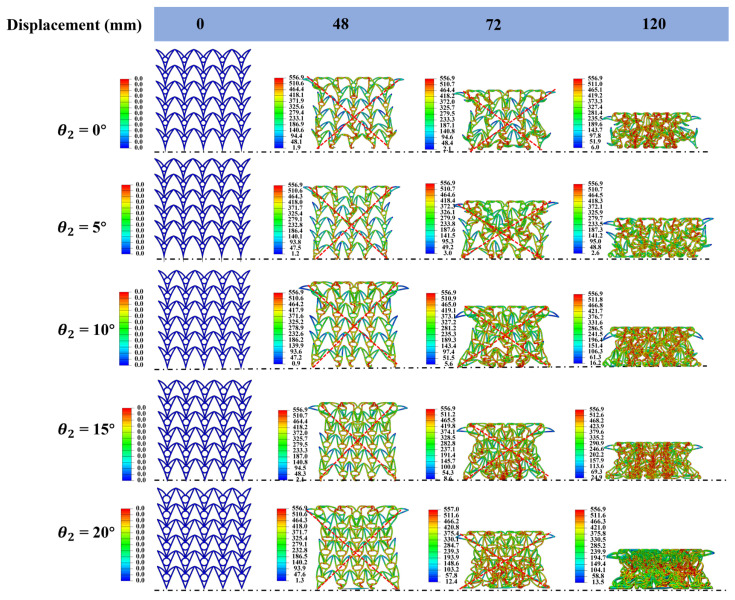
Deformation patterns of honeycomb structures with different $\theta_{2}$ values under quasi-static compression (the stress unit in this figure is MPa).

Honeycomb samples with different *θ*_2_ values (0°, 5°, 10°, 15°, and 20°) were designed. To isolate the effect, $\theta_{1}$, *d*, and *N*_x_ × *N*_z_ were fixed at 30°, 10 mm, and 6 × 5, respectively, for all samples. The macroscopic dimensions in the x-z plane were 180 × 195 mm. Figure 14 summarizes the mechanical performance for different *θ*_2_ values. Figure 15 presents the deformation patterns for different *θ*_2_ values under quasi-static compression. At a compressive displacement of 48 mm, all structures develop a characteristic X-shaped deformation band, indicating that the deformation pattern at this stage is only weakly affected by *θ*_2_. However, the force–displacement responses differ markedly among the configurations. Geometric and density analyses suggest that these differences arise from two effects at small *θ*_2_ (e.g., 5°). First, a small *θ*_2_ reduces the transverse width in the lower part of the cell, lowering lateral sway resistance and increasing susceptibility to global instability [63]. Second, reducing *θ*_2_ sparsifies the material distribution in the cell walls, decreases the relative density, and further undermines load-bearing stability.

Regarding performance metrics, as *θ*_2_ increases from 0° to 20°, both the peak crushing force (PCF) and average bearing force (ABF) increase. Specifically, PCF rises from 47.09 kN to 54.77 kN, and ABF rises from 52.45 kN to 67.11 kN. Notably, to maintain constant cell dimensions in both the transverse and longitudinal directions, increasing *θ*_2_ requires a concomitant increase in the radius of the central circular feature. This geometric adjustment likely drives the increase in PCF because a larger-radius circular feature provides stronger elastic support, alleviates local stress concentrations during compression, and increases the initial peak load. Inspection of the force–displacement curves in Figure 14a indicates that the plateau force generally increases with *θ*_2_, which appears positively correlated with the circular-feature radius. Specifically, larger *θ*_2_ produces a stronger reaction force from the circular feature on the cell walls, enhancing the overall load-carrying capacity and increasing ABF.

In terms of deformation stability and SEA, at *θ*_2_ = 5°, the structure exhibits pronounced global distortion during compression, accompanied by severe twisting of the cell beams. As *θ*_2_ increases, this nonuniform deformation is substantially mitigated. When *θ*_2_ exceeds about 20°, the increased inclination of the lower curved beam reduces the axial load-bearing component and amplifies bending deformation. Consequently, localized instability and premature plastic hinge formation occur, leading to structural collapse. Two atypical deformation modes—global twisting at *θ*_2_ = 5° and late-stage collapse at *θ*_2_ = 20°—obscure the systematic relationship between SEA and *θ*_2_. Excluding these two outliers reveals a clear increase in SEA with *θ*_2_. Overall, *θ*_2_ = 10–15° offers a favorable trade-off: it preserves a stable NPR response while improving deformation stability and energy absorption efficiency.

Although the present parametric study considers a limited range of cell numbers, the deformation mechanisms observed in this study are primarily governed by the local geometry of the unit cell. Therefore, the mechanical response of larger structures is expected to exhibit similar progressive deformation patterns, provided that the global boundary effects remain limited. As the number of cells increases, the overall response tends to approach a stable macroscopic behavior dominated by the repeating unit cell topology [64]. Consequently, the trends identified in this study are expected to remain applicable to larger honeycomb assemblies.

## 4. Multi-Objective Optimization

The FE simulation results indicate that different structural parameters distinctly affect the mechanical response of the proposed design. Therefore, performance cannot be reliably characterized using only a few “representative” parameter values, and the parameter–performance relationships may be nonlinear. In energy-absorbing structures, SEA and PCF often conflict [65]. Accordingly, a multi-objective optimization problem is formulated by selecting the cell angles and thickness as design variables and SEA and PCF as objective functions, and the Pareto-optimal set is obtained [66]. The aim is to optimize key parameters of the proposed structure to identify configurations with improved overall performance. ABF is often strongly correlated with SEA and can be obtained by integrating the force–displacement curve. Therefore, ABF is typically treated as a constraint or a post hoc metric rather than a standalone optimization objective.

### 4.1. Design of Experiments

In this study, Optimal Latin Hypercube Sampling (OLHS) was adopted. Starting from an initial Latin Hypercube Sampling (LHS) design [67], the sampling plan was iteratively refined by maximizing the minimum pairwise distance among sample points, thereby improving space-filling uniformity in the multidimensional design space. This improved uniformity is critical for accurate surrogate model construction, particularly for nonlinear response surfaces. A design matrix of 50 OLHS sample points was generated. Using the same model settings as in the compression experiments, the objective responses were computed at each sample point. Table 1 lists the response values for the 50 sample points.

### 4.2. Surrogate Model Design

After collecting a sufficient dataset, a surrogate model was constructed to efficiently and accurately approximate the nonlinear mapping between the design variables and the objective responses (SEA and PCF). A radial basis function (RBF) method was selected to construct surrogate models linking the design variables to the optimization objectives [68]. In RBF modeling, the kernel function strongly influences model performance. Because the design space is continuous and the objectives are expected to vary smoothly with the design variables, a linear kernel was adopted to promote parsimony and computational efficiency and to reduce overfitting risk. The resulting RBF model is written as follows:

(11) $$\hat{y}(x)=\sum_{i=1}^{N}\lambda_{i}\phi (\|x-x_{i}\|)+p(x)$$

where N is the number of samples, $y_{i}$ and ${\hat{y}}_{i}$ denote the responses obtained from the FE simulations and the surrogate model predictions, respectively, *λ_i_* denotes the weight coefficient, **p**(***x***) is the polynomial trend term, and the linear kernel is given by *ϕ(r) = r*. Using the 50 OLHS samples, two independent RBF surrogate models were trained to predict SEA and PCF, respectively.

### 4.3. NSGA-II Multi-Objective Optimization

The second-generation non-dominated sorting genetic algorithm (NSGA-II) was used to solve the RBF surrogate model [69]. The resulting multi-objective optimization problem is summarized as follows:

(12) $$\begin{array}{cc} \mathrm{Maximize}\;\mathrm{SEA} & \\ \mathrm{Minimize}\;\mathrm{PCF} & \\ s.t.\left\{\begin{array}{c} 20.00{}^{\circ}\leq \theta_{1}\leq 60.00{}^{\circ}\\ 5.00{}^{\circ}\leq \theta_{1}\leq 20.00{}^{\circ}\\ 2.00\mathrm{mm}\leq d\leq 20.00\mathrm{mm} \end{array}\right. & \end{array}$$

In NSGA-II, candidate solutions were screened according to predefined feasibility criteria, and only designs satisfying the geometric and performance constraints were retained for Pareto sorting. The population size was set to 300, and the maximum number of generations was 100. Tournament selection was adopted for parent selection. The crossover fraction was set to 0.9, and the mutation operator used an adaptive feasible mutation strategy. NSGA-II produced a well-distributed Pareto front with corresponding objective values, highlighting the trade-off between SEA and PCF.

Furthermore, the predictive capability of the surrogate model was evaluated using several statistical indicators, including the coefficient of determination (R^2^), root mean square error (RMSE), mean absolute error (MAE), and leave-one-out cross-validation (LOOCV) error, as summarized in Table S1.

The coefficient of determination is defined as:

(13) $$R^{2}=1-\frac{\sum_{i=1}^{N}{\left(y_{i}-{\hat{y}}_{i}\right)}^{2}}{\sum_{i=1}^{N}{\left(y_{i}-{\bar{y}}_{i}\right)}^{2}}$$

where $\bar{y}$ represents the mean value of the simulation results.

The RMSE is expressed as:

(14) $$\mathrm{RMSE}=\sqrt{\frac{1}{N}\sum_{i=1}^{N}{\left(y_{i}-{\hat{y}}_{i}\right)}^{2}}$$

The MAE is defined as:

(15) $$\mathrm{MAE}=\frac{1}{N}\sum_{i=1}^{N}\left|y_{i}-{\hat{y}}_{i}\right|$$

To further evaluate the generalization capability of the surrogate model, the LOOCV method was employed. In this approach, one sample is removed from the dataset, and the surrogate model is reconstructed using the remaining samples. The excluded sample is then predicted using the reconstructed model.

The LOOCV error can be calculated as:

(16) $${\mathrm{RMSE}}_{\mathrm{LOOCV}}=\sqrt{\frac{1}{N}\sum_{i=1}^{N}{\left(y_{i}-y_{-i}\right)}^{2}}$$

where $y_{i}$ represents the FE simulation result of the *i*-th sample and ${\hat{y}}_{-i}$ denotes the predicted value obtained from the surrogate model constructed without the *i*-th sample.

The predictive capability of the constructed RBF surrogate model was evaluated using several statistical indicators, including R^2^, RMSE, MAE, and LOOCV error. As shown in Table 3, the surrogate model achieved R^2^ values of 0.97 and 0.95 for SEA and PCF, respectively. The relatively small RMSE and MAE values indicate high prediction accuracy. Moreover, the LOOCV errors are close to the corresponding training errors, suggesting good generalization capability of the model. The comparison between the predicted and FE results further confirms the strong agreement between the surrogate model and numerical simulations, demonstrating that the developed model is sufficiently accurate for subsequent multi-objective optimization.

**Figure 16:**
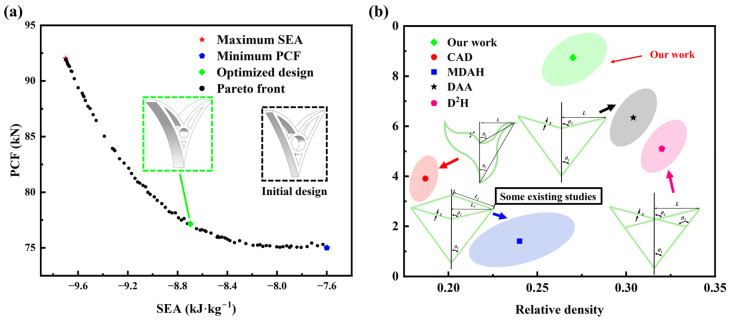
Optimization results and comparison with other studies: (**a**) Pareto front in multi-objective optimization; (**b**) SEA-based Ashby plot and the corresponding unit cell [28,29,69,70].

Figure 16a shows the Pareto solution set. The solutions with the maximum SEA and the minimum PCF coincide with the final results of the corresponding single-objective optimizations. To select a single design for subsequent validation, a satisfaction function was used to rank the Pareto-optimal solutions. Because SEA and PCF are conflicting objectives, they cannot be simultaneously optimized. Therefore, the satisfaction value of each Pareto-optimal solution was computed using Equation (17), and the solution with the minimum value was selected as the final design for each optimization algorithm.

(17) $$V=\frac{\mathrm{SEA}-{\mathrm{SEA}}_{\min}}{{\mathrm{SEA}}_{\max}-{\mathrm{SEA}}_{\min}}+\frac{\mathrm{PCF}-{\mathrm{PCF}}_{\min}}{{\mathrm{PCF}}_{\max}-{\mathrm{PCF}}_{\min}}$$

where SEA_max_ and SEA_min_ are the maximum and minimum SEA values within the Pareto set, respectively, and PCF_max_ and PCF_min_ are defined analogously for PCF.

The selected optimal parameter combinations and their corresponding predicted responses are listed in Table 4. The optimized design parameter combinations and their predicted SEA and PCF performance were then simulated and comparatively analyzed against the baseline design; the results are summarized in Table 2. The results show that the optimized design improves SEA while limiting the increase in PCF, supporting the effectiveness of the proposed optimization approach. Figure 16b illustrates the influence of relative density on the specific energy absorption (SEA) of different structures. While maintaining a relatively low density, this design achieves a high SEA, highlighting its efficient energy absorption capability.

## 5. Conclusions

This study addresses the low energy absorption efficiency and limited deformation stability of conventional double-arrow honeycombs (DAHs) by proposing a curvilinear hybrid auxetic honeycomb (CHAH) enabled by geometric and topological innovations. By integrating geometric design, finite element simulations, and performance optimization, the mechanical response and energy dissipation mechanisms are elucidated, and a parametric design and optimization framework is established. The main conclusions are as follows.

1. A curvilinear double-arrow honeycomb with a negative Poisson’s ratio (CHAH) is proposed to enhance energy absorption and promote uniform deformation in DAH under large compressive strains. Using a local substitution and component addition strategy, straight cell walls are replaced by continuously curved beams. A circular supporting subcell with a positive Poisson’s ratio is embedded, forming a coupled curved-beam–circular cooperative deformation system. The key design parameters are angle *θ*_1_, angle *θ*_2_, and thickness *d*, which together enable tunable stiffness and energy absorption performance.
2. The geometric parameters of the CHAH strongly govern its effective properties and Poisson’s ratio response. A pronounced negative Poisson’s ratio effect is observed across all investigated configurations. The structural stiffness decreases with *θ*_1_ and increases with *θ*_2_. When *θ*_1_ approaches *θ*_2_, the load transfer path shortens, resulting in a pronounced increase in stiffness. In addition, both PCF and ABF increase approximately linearly with d, while SEA remains essentially unchanged.
3. At the same relative density and using identical material, the CHAH consistently outperforms the DAH in energy absorption performance. Specifically, the CHAH achieves a maximum increase in specific energy absorption (SEA) of 67.06% relative to the DAH, demonstrating the advantage of the proposed configuration for lightweight crashworthy design.
4. To further improve the CHAH, multi-objective optimization using NSGA-II was performed on an RBF-based surrogate model to capture the trade-off between SEA and PCF. Feasible solutions formed a well-distributed Pareto front. A single compromise design was then selected using a satisfaction function. Relative to the initial CHAH, the selected design provides an additional 2.3% improvement in SEA while limiting the increase in PCF, supporting the effectiveness of the proposed optimization strategy.

Overall, the CHAH is a promising negative Poisson’s ratio (NPR) honeycomb for energy absorption under quasi-static compression, offering high SEA, a stable plateau response, and tunable stiffness. Since the present study focuses on quasi-static compression, the influence of strain rate effects on deformation modes and energy absorption has not been considered. In practical crash or impact scenarios, rate-dependent material behavior may affect the plateau response and peak crushing force. Future work will therefore include studies of rate-dependent behavior, including impact loading, to strengthen the integrated theory–simulation–experiment validation framework and facilitate practical implementation in protective components.

## Figures and Tables

**Table 1:** Definition and ranges of the geometric parameters of the CHAH structure.

Parameter	Symbol	Range	Unit
Upper ligament angle	*θ*_1_	20–60	°
Lower ligament angle	*θ*_2_	0–20	°
Upper curvature radius	*R*_1_	180	mm
Lower curvature radius	*R*_2_	30	mm
Circular subcell diameter	*D*	Depends on *θ*_1_ and *θ*_2_	mm
Out-of-plane thickness	*d*	10–30	mm
Cell wall thickness	*L*	2	mm
Number of cells in x-direction	*N*_x_	5	quantity
Number of cells in z-direction	*N*_z_	1–6	quantity

**Table 2:** Material properties of base materials.

Material	Density (g/cm^3^)	Young’s Modulus (GPa)	Poisson’s Ratio	Yield Stress (MPa)
316 L steel	7.85	168	0.29	556

**Table 3:** Accuracy metrics of the RBF surrogate model.

Response	R^2^	RMSE	MAE	LOOCV RMSE
SEA (kJ·kg^−1^)	0.97	0.42	0.31	0.48
PCF (kN)	0.95	3.26	2.14	3.71

**Table 4:** Results and validation of the multi-objective optimization.

Model	*θ*_1_ (°)	*θ*_2_ (°)	*d* (mm)	SEA (kJ·kg^−1^)	PCF (kN)
Initial model	30.0	15.0	10.0	8.54	53.41
Optimization model	25.8	5.0	19.0	8.70	77.15
Simulation verified model	25.8	5.0	19.0	8.74	78.65

## Data Availability

The original contributions presented in this study are included in the article/Supplementary Materials. Further inquiries can be directed to the corresponding author.

## Supplementary Materials

The following supporting information can be downloaded at https://www.mdpi.com/article/10.3390/ma19091791/s1: Table S1. The objective response values at the sampled points.
