# Supplementary material for: Energy Absorption of Curvilinear Hybrid Auxetic Honeycombs
Source: Materials (Basel). 2026 Apr 28;19(9):1791. doi: 10.3390/ma19091791 (PMC13165456; doi:10.3390/ma19091791)
Supplement: Supplementary file 1 [file materials-19-01791-s001.zip › materials-4252217-supplementary.pdf]

Supplementary Materials.

Table S1. The objective response values at the sampled points.

	θ_1/°	θ_2/°	d/mm	SEA/kJ·kg^{-1}	PCF/kN
1	36.5	9.2	19.1	7.5	101.1
2	51.5	16.3	25.9	7.4	139.8
3	28.0	15.8	23.1	7.7	128.1
4	51.1	13.4	16.3	7.4	85.27
5	44.8	12.6	18.7	7.0	98.7
6	40.8	17.1	19.8	5.5	107.7
7	54.4	5.1	17.3	5.8	94.7
8	20.1	9.6	23.8	6.9	112.6
9	42.4	6.1	18.3	8.7	97.4
10	55.4	16.9	18.3	7.0	94.8
11	49.3	14.6	20.2	6.8	106.3
12	47.5	7.4	26.5	5.9	142.3
13	54.4	19.1	25.6	5.7	132.4
14	49.8	15.4	28.3	7.5	151.5
15	39.2	7.0	20.0	7.9	101.6
16	43.1	9.0	22.8	3.9	122.0
17	58.1	8.3	16.9	5.1	90.3
18	26.1	7.6	17.5	8.0	85.4
19	48.6	16.6	15.2	6.7	83.5
20	33.1	19.4	21.8	7.7	122.7
21	58.8	17.9	22.1	6.3	118.0
22	40.0	10.2	29.1	7.0	151.7
23	34.1	8.6	29.4	6.5	152.3
24	28.1	11.2	25.0	7.9	128.7
25	26.8	18.2	23.1	7.7	132.3
26	57.0	12.2	16.0	6.9	84.3
27	43.2	18.5	21.3	6.1	115.5
28	20.9	13.8	28.1	6.7	150.8
29	46.0	11.9	15.5	7.4	81.7
30	23.2	18.1	29.5	8.1	171.0
31	22.5	19.9	17.9	7.2	104.9
32	45.1	8.7	28.6	8.5	154.5
33	37.6	14.1	24.9	7.9	136.8
34	59.3	5.4	24.5	5.3	134.3
35	32.1	9.8	24.0	7.8	124.5
36	31.6	5.7	20.6	8.4	101.7
37	35.0	17.4	27.7	9.2	156.9
38	46.5	13.7	27.0	6.8	143.6
39	30.6	6.4	25.4	7.7	126.9
40	22.0	16.1	16.8	6.6	93.4
41	37.9	19.3	19.4	8.4	117.7
42	52.7	11.6	21.0	8.6	151.7
43	25.6	13.1	22.4	6.5	152.3
44	53.5	7.7	23.7	9.2	128.7
45	56.0	10.8	26.2	6.6	132.3
46	24.0	10.6	15.6	6.3	84.3
47	29.1	14.4	29.8	6.7	115.5
48	39.3	11.8	21.4	8.3	150.8
49	30.0	15.1	26.8	6.7	81.7
50	35.5	6.7	27.6	6.8	171.0
